# Inhibition of PTGS1 promotes osteogenic differentiation of adipose-derived stem cells by suppressing NF-kB signaling

**DOI:** 10.1186/s13287-019-1167-3

**Published:** 2019-02-13

**Authors:** Yuejun Wang, Yunsong Liu, Min Zhang, Longwei Lv, Xiao Zhang, Ping Zhang, Yongsheng Zhou

**Affiliations:** 1 Department of Prosthodontics, Peking University School and Hospital of Stomatology, 22 Zhongguancun South Avenue, Haidian District, Beijing, 100081 China; 2 National Engineering Lab for Digital and Material Technology of Stomatology, Peking University School and Hospital of Stomatology, Beijing, 100081 China

**Keywords:** PTGS1, NF-κB, Osteogenic differentiation, ASCs

## Abstract

**Background:**

Tissue inflammation is an important problem in the field of human adipose-derived stem cell (ASC)-based therapeutic bone regeneration. Many studies indicate that inflammatory cytokines are disadvantageous for osteogenic differentiation and bone formation. Therefore, overcoming inflammation would be greatly beneficial in promoting ASC-mediated bone regeneration. The present study aims to investigate the potential anti-inflammatory role of Prostaglandin G/H synthase 1 (PTGS1) during the osteogenic differentiation of ASCs.

**Methods:**

We performed TNFα treatment to investigate the response of PTGS1 to inflammation. Loss- and gain-of-function experiments were applied to investigate the function of PTGS1 in the osteogenic differentiation of ASCs ex vivo and in vivo. Western blot and confocal analyses were used to determine the molecular mechanism of PTGS1-regulated osteogenic differentiation.

**Results:**

Our work demonstrates that PTGS1 expression is significantly increased upon inflammatory cytokine treatment. Both ex vivo and in vivo studies indicate that PTGS1 is required for the osteogenic differentiation of ASCs. Mechanistically, we show that PTGS1 regulates osteogenesis of ASCs via modulating the NF-κB signaling pathway.

**Conclusions:**

Collectively, this work confirms that the PTGS1-NF-κB signaling pathway is a novel molecular target for ASC-mediated regenerative medicine.

**Electronic supplementary material:**

The online version of this article (10.1186/s13287-019-1167-3) contains supplementary material, which is available to authorized users.

## Background

Traditional treatments for bone augmentation using autologous or allogeneic bone graft, bone substitute material, or guided bone regeneration technology often show limited clinical benefit [1]. In addition, most therapeutic bone defects or causes of bone loss, such as periodontitis, traumatic bone injury, or insufficient bone mass around implants, are usually accompanied by inflammation. The above methods fail to control the inflammatory microenvironment. Current advances in bone tissue engineering hold significant future for regenerating bone tissue. Considering their capabilities of self-renewal, proliferation, multiple differentiation, and abundant availability, human adipose-derived stem cells (ASCs) are an ideal origin of adult mesenchymal stem cells (MSCs) for bone tissue regeneration [2–4]. Therefore, our focus herein is to determine how to vigorously activate the osteogenesis capability and inflammatory regulation potential of ASCs and to identify key parameters of a stem cell-based approach for bone tissue regeneration.

Prostaglandin G/H synthase 1 (PTGS1) is an enzyme of prostaglandin synthesis that is of great physiological significance. The promoter region of PTGS1 is GC-rich and has multiple transcription factor binding sites, but lacks functional TATA and CAAT sequences. As an inherent housekeeping enzyme, PTGS1 is mainly expressed in the stomach, kidney, and platelets, and its fundamental functions include regulating the resistance of peripheral vasculature, maintenance of renal blood flow, protection of gastric mucosa, and modulation of platelet aggregation. PTGS1 is also perceived as acting critical roles on pathophysiological progress of inflammation, arthritic disease, and cancer [5–10]. Clinical studies have shown that as a specific inhibitor of PTGS1, low-dose aspirin can effectively exert antipyretic, analgesic, and anti-inflammatory functions; these are reported to prevent or treat numerous associated diseases [11]. Importantly, it has been reported that application of PTGS1 inhibitors or silencing of *PTGS1* expression greatly attenuates the inflammatory response induced by LPS via negatively governing the nuclear factor kappa B (NF-κB) signaling pathway [12, 13]. In addition, previous studies have indicated PTGS1 is involved in the differentiation of macrophages and monocytes [14]. Another PTGS1 inhibitor, BGJb, has been found to inhibit bone resorption [15]. However, the role of PTGS1 in the osteogenic differentiation of ASCs and its potential role in the regulation of inflammation have not been reported.

Bone remodeling is a constant homeostasis that is frequently disturbed by pro-inflammatory cytokines which could curb bone formation and lead to bone loss [16, 17]. NF-κB is a core transcription factor that governs osteogenesis and inflammatory response in MSCs. Significant evidence has accumulated implying the strong potential of NF-κB as a therapeutic target for treating inflammation-associated bone remodeling [18, 19]. In this study, we aimed to evaluate the role of PTGS1 in the osteogenic differentiation and inflammatory regulation of human ASCs. Our results demonstrate that deletion of PTGS1 greatly promotes the osteogenesis of ASCs ex vivo and in vivo and depletion of PTGS1 possesses potential anti-inflammatory function via repressing NF-κB pathway, suggesting the potential utility of PTGS1 in ASC-based bone tissue engineering.

## Methods

### Cell cultures and osteogenic induction

Primary human ASCs from three donors (Batch number 2249, 11537, and 19382) were purchased from the ScienCell Research Laboratories (Carlsbad, CA, USA; catalogue number 7510). ASCs were cultured in a humidified incubator at 37 °C under 5% CO_2_ in the DMEM alpha modified Eagle’s medium (Invitrogen, Carlsbad, CA, USA), supplemented with 10% (*v*/*v*) fetal bovine serum, 100 U/mL penicillin, 2 mmol/L glutamine, and 100 μg/mL streptomycin (Invitrogen). For TNFα (R&D Systems, Minneapolis, MN, USA) treatment, ASCs were synchronized by starvation for 24 h in culture medium without fetal bovine serum, then changed to ordinary medium with TNFα treatment.

For osteogenic differentiation induction, cells were cultured in osteogenic induction medium consisting of DMEM alpha modified Eagle’s medium with 10% (*v*/*v*) FBS, 100 U/mL penicillin, 100 μg/mL streptomycin, 10 mM β-glycerophosphate, 0.2 mM l-ascorbic acid, and 100 nM dexamethasone.

### Lentivirus infection

Recombinant lentivirus targeting *PTGS1* was purchased from the GenePharma company. The study was performed as described previously [20–22]. For viral infection, ASCs were cultured overnight, infected with lentivirus with 4 μg/mL polybrene (Sigma-Aldrich, St. Louis, MO, USA) for 8 h, and then cultured with an ordinary medium. After 96 h, 1 mg/mL puromycin (Sigma-Aldrich) was added into the medium to select the infected cells. The shRNA sequences were as follows: NC, TTCTCCGAACGTGTCACGT; *PTGS1*sh1, GATCCAGAACAGTGGCTCG; and *PTGS1*sh2, AAGTGCCATCCAAACTCTATCTT.

For gene overexpression, a lentivirus expressing PTGS1 was purchased from the Cyagen company and used for ASC infection.

### ALP activity and ALP staining

For alkaline phosphatase (ALP) activity, ASCs were cultured in osteogenic induction medium. After 5 days, 5 μL protein lysate was extracted and then tested following an ALP activity kit (Sigma-Aldrich). Signal was normalized by protein concentration.

After 1-week osteogenic induction, cells were fixed in 4% paraformaldehyde for 15 min. Then cells were stained with a BCIP/NBT staining kit (CWBIO, Beijing, China) at room temperature for 10 min.

### Alizarin red staining and quantification

After osteogenic induction for 2 weeks, cells were fixed in 95% ethanol and then incubated with 2% Alizarin red buffer (Sigma-Aldrich). To exam the level of calcium, the coloration was destained and the absorbance of calcium was measured on a multiplate reader at 562 nm. The final concentration of calcium was normalized to protein concentration of each plate.

### Real-time reverse transcriptase-polymerase chain reaction

Total cellular RNA was extracted from ASCs using Trizol reagent (Invitrogen). We synthesized cDNA from 1 μg RNA aliquots. Real-time reverse transcriptase-polymerase chain reaction (RT-qPCR) was performed with Power SYBR Green PCR Master Mix (Roche, Basel, Switzerland) and tested by a 7500 Real-Time PCR Detection System (Applied Biosystems, Thermo Fisher, MA, USA). GAPDH was used as the internal control. The primer sequences were as follows: *GAPDH* (forward) 5′-CGGACCAATACGACCAAATCCG-3′ and (reverse) 5′-AGCCACATCGCTCAGACACC-3′; *PTGS1* (forward) 5′-CAATGCCACCTTCATCCGAG-3′ and (reverse) 5′-GATAAGGTTGGAGCGCACTG-3′; *ALP* (forward) 5′-GACCTCCTCGGAAGACACTC-3′ and (reverse) 5′-TGAAGGGCTTCTTGTCTGTG-3′; *OCN* (forward) 5′-AGCAAAGGTGCAGCCTTTGT-3′ and (reverse) 5′-GCGCCTGGGTCTCTTCACT-3′; *BSP* (forward) 5′-CAGGCCACGATATTATCTTTACA-3′ and (reverse) 5′-CTCCTCTTCTTCCTCCTCCTC-3′; *RUNX2* (forward) 5′-TCTTAGAACAAATTCTGCCCTTT-3′ and (reverse) 5′-TGCTTTGGTCTTGAAATCACA-3′; *OSX* (forward) 5′-CCTCCTCAGCTCACCTTCTC-3′ and (reverse) 5′-GTTGGGAGCCCAAATAGAAA-3′; *IL6* (forward) 5′-CGCAACAACTCATCTCATTCTGCG-3′ and (reverse) 5′-CATGCTACATTTGCCGAAGAGC-3′; *IL8* (forward) 5′-CGGATAAAGGGCCAAGAGAATATCCG-3′ and (reverse) 5′-TCACATTCTAGCAAACCCATTCAA-3′; and *SELE* (forward) 5′-AGCTTCCCATGGAACACAAC-3′ and (reverse) 5′-CTGGGCTCCCATTAGTTCAA-3′. Relative gene expression is calculated by using the 2^−ΔΔCt^ method.

### Nuclear and cytoplasmic extraction

Cells were suspended and swollen in buffer A (10 mM HEPES, 0.1 mM EDTA, 1 mM DTT, 0.1 mM EGTA, 10 mM KCl, 0.15% NP-40, and 1:100 proteinase inhibitor cocktail) on ice for 10 min. After centrifugation, the supernatant of the sample was collected as the cytoplasmic extract. The rest of pellet was washed with PBS and then resuspended in buffer B (20 mM HEPES, 1 mM EGTA, 1 mM EDTA, 1 mM DTT, 400 mM NaCl, 0.5% NP-40, and 1:100 proteinase inhibitor cocktail) at 4 °C for 25 min. After centrifugation, the supernatant was collected and used as the nuclear extract.

### Western blot experiment

ASCs were lysed in the RIPA buffer (10 mM Tris-HCl, 1% sodium dodecyl sulfate, 50 mM sodium fluoride, 50 mM β-glycerophosphate, 1 mM EDTA, 1% NP-40, and 1:100 proteinase inhibitor cocktail). Thirty-microgram protein of each sample was used for analysis as described previously [20–22]. Primary antibodies against PTGS1, GAPDH, p-IκBα (ser32/ser36), p-p65 (Ser536), p65, tubulin, PCAF (Cell Signaling Technology), and IκBα (Abcam) were diluted 1:1500 and then incubated with the membrane overnight at 4 °C. Horseradish peroxidase-conjugated anti-mouse or anti-rabbit secondary antibodies (Cell Signaling Technology) were diluted 1:10,000 and incubated with the membrane for 1 h, and the membrane was visualized. Next, band intensity was quantified by using the Image J software, and target band signal was normalized to the corresponding internal control (GAPDH, PCAF, or tubulin).

### Immunofluorescence staining

Firstly, cells that grew on glass coverslips were fixed and then permeabilized with 0.25% Triton X-100 for 10 min, blocked with 0.8% BSA for 1 h at room temperature. Next, cells were incubated with the primary antibody against p65 (Cell Signaling Technology) overnight at 4 °C and treated with appropriate secondary antibody. The nucleus was counterstained with DAPI. The experiment was performed as described previously [20, 21].

### Analyses of bone formation in vivo

This study was approved by the Institutional Animal Care and Use Committee of the Peking University Health Science Center (LA2014233), and all related experiments were performed in accordance with the Institutional Animal Guidelines. In vivo study was performed as described previously [20, 21]. The third passage of ASCs that transfected with target gene was cultured in a proliferation medium before implantation. For each implantation site, 2 × 10^6^ cells were mixed with 40-mg synthograft (Bicon) at 37 °C for 1 h, then the mixture pellet was acquired and implanted on the dorsal subcutaneous site of homozygous nude mice (6-week-old BALB/c, *n* = 10). After 8 weeks, the mice were euthanized by CO_2_ asphyxiation. Next, specimens were harvested and then fixed, followed by decalcification in 10% EDTA buffer (pH 7.4) for 1.5 weeks, then dehydrated, and embedded. Tissue section (thickness 5 μm) was cut and then stained with HE, Masson’s trichrome, and immunohistochemical staining (primary antibody against OCN (Abcam)). To quantify the bone-like tissue, 15 images from each sample were randomly collected, and the area of new bone formation versus total area was calculated by the SPOT 4.0 software (Diagnostic Instruments, Sterling Heights, MI, USA).

### Statistics

All statistical calculations were performed by using SPSS10 statistical software. Comparisons between two groups were analyzed by independent two-tailed Student’s *t* tests, and comparisons between more than two groups were analyzed by one-way ANOVA followed by Tukey’s post hoc test. Data presented are derived from three ASC strains. All data are showed as the mean ± standard deviation (SD) of three to ten experiments per group. *P* value < 0.05 was considered statistically significant.

## Results

### PTGS1 expression is stimulated by TNFα treatment

**Fig. 1:**
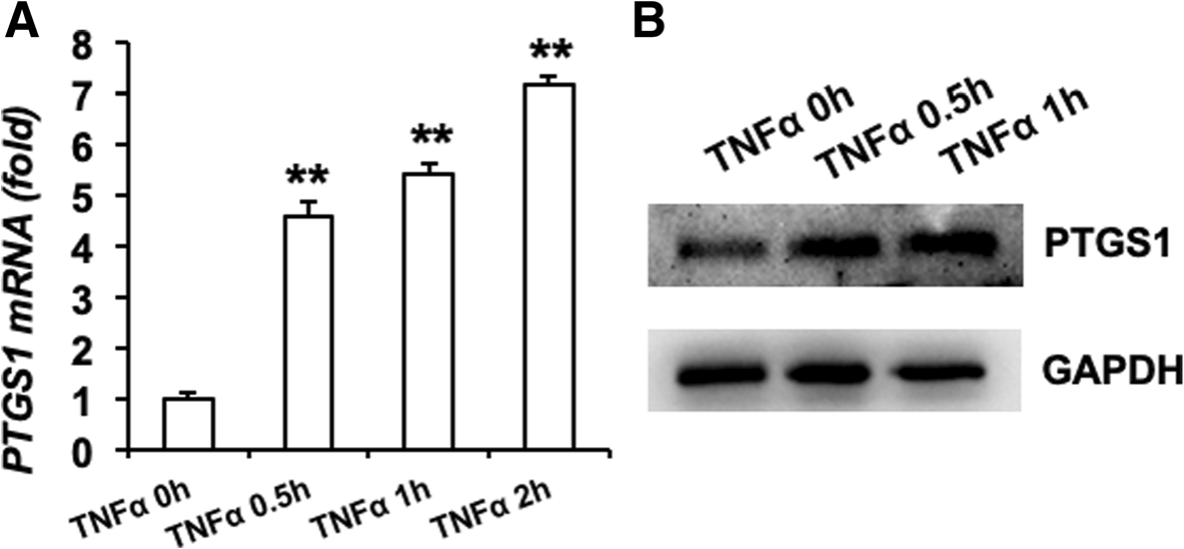
PTGS1 expression is stimulated by TNFα treatment. **a**, **b** PTGS1 expression was significantly induced when treated with TNFα for 0.5, 1, and 2 h as determined by real-time RT-qPCR and Western blot analysis. GAPDH was used as an internal control. All data are shown as the mean ± SD, *n* = 3. **P < 0.01. h hour

First, to evaluate the potential response of PTGS1 during inflammation, we investigated the expression of PTGS1 in ASCs with inflammation factor TNFα treatment. As shown in Fig. 1a, b, real-time RT-qPCR and Western blot analyses showed significantly increased expression of PTGS1 upon TNFα treatment.

### PTGS1 is required for the osteogenic differentiation of ASCs in vitro

**Fig. 2:**
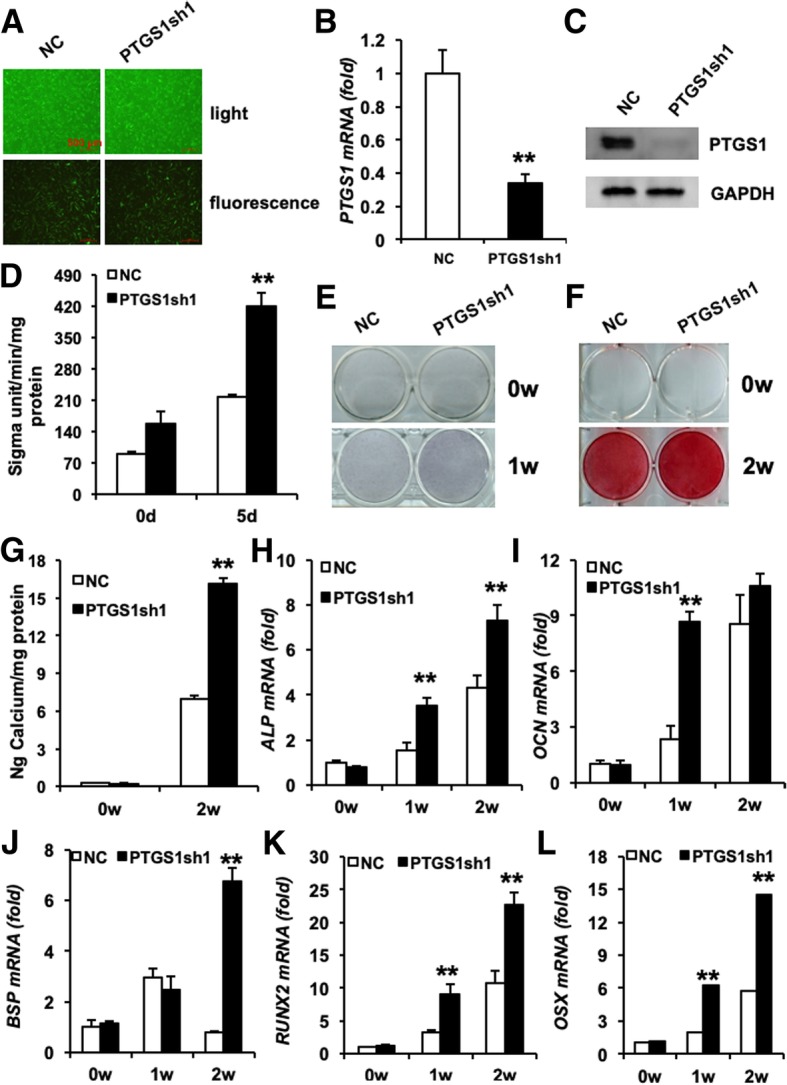
Knockdown of PTGS1 enhances the osteogenic differentiation of ASCs in vitro. **a** GFP-positive ASCs under light and fluorescence microscopy. Scale bar, 500 μm. **b**, **c** Knockdown of PTGS1 was verified by real-time RT-qPCR and Western blot. **d**, **e** PTGS1 silencing increased the ALP activity and ALP staining in ASCs. **f**, **g** PTGS1 knockdown increased mineralization, as shown by Alizarin red staining and quantitative calcium analysis*.* **h**–**l** The mRNA expressions of *ALP*, *OCN*, *BSP*, *RUNX2*, and *OSX* were determined by RT-qPCR. GAPDH was used as an internal control. All data are shown as the mean ± SD, *n* = 3. **P < 0.01. NC negative control cells, *PTGS1*sh PTGS1 knockdown cells, d day, w week

To investigate the function of PTGS1 during the osteogenic differentiation of ASCs, we established *PTGS1* stable knockdown cells. Fluorescent staining detecting GFP showed that the majority of ASCs were infected with lentivirus (Fig. 2a), and silencing efficiency was further measured by real-time RT-qPCR (Fig. 2b) and Western blot (Fig. 2c). The infected cells were then cultured in an osteogenic induction medium to explore osteogenic differentiation capacity. The results revealed that silencing of PTGS1 strongly increased both ALP activity and staining, as shown in Fig. 2d, e. In addition, *PTGS1*sh cells had elevated mineralization deposition as shown by Alizarin red staining (Fig. 2f) and quantitative calcium analysis (Fig. 2g). RT-qPCR results indicated that knockdown of PTGS1 enhanced the expression of osteogenesis-related genes such as alkaline phosphatase (*ALP*), osteocalcin (*OCN*), bone sialoprotein (*BSP*), runt-related transcription factor 2 (*RUNX2*), and osterix (*OSX*) at 0, 1, and 2 weeks after induction (Fig. 2h–l).

In order to avoid off-target effect, an additional sequence targeting *PTGS1* was used. After transfection, results indicated that the efficiency of infection was greater than 80% (Additional file 1: Figure S1A-C). Consistently, PTGS1 knockdown promoted osteogenic differentiation as revealed by ALP activity, ALP staining, Alizarin red staining, and quantitative calcium analysis (Additional file 1: Figure S1D-G). Moreover, real-time RT-qPCR results showed upregulation of related genes (*ALP*, *OCN*, *BSP*, *RUNX2*, and *OSX*) (Additional file 1: Figure S1H-L) in *PTGS1*sh cells. Taken together, these data demonstrate that PTGS1 plays a critical function in the osteogenic differentiation of ASCs.

**Fig. 3:**
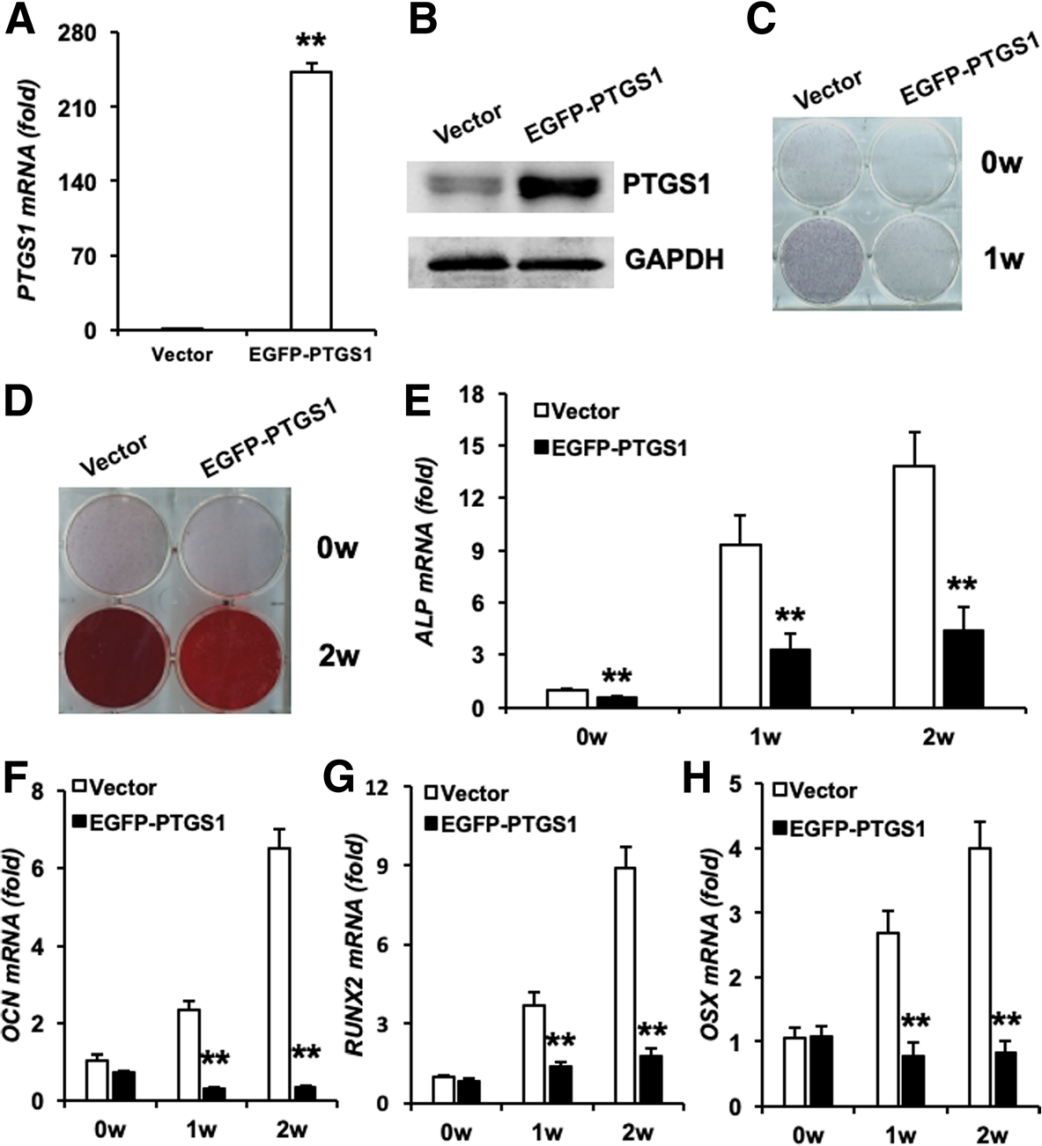
PTGS1 overexpression inhibits the osteogenic differentiation of ASCs in vitro*.* **a**, **b** EGFP-PTGS1-infected ASCs showed overexpression of PTGS1, as shown by real-time RT-qPCR and Western blot. **c** PTGS1 overexpression reduced ALP staining. **d** PTGS1 overexpression decreased mineralization, as shown by Alizarin red staining. **e**–**h** Real-time RT-qPCR analysis of the expression of *ALP*, *OCN*, *RUNX2*, and *OSX.* GAPDH was used as an internal control. All data are shown as the mean ± SD, *n* = 3. **P < 0.01. w week

Next, we used ectopic *PTGS1* lentivirus to infect ASCs, and PTGS1 overexpression was confirmed by RT-qPCR and Western blot (Fig. 3a, b). As shown in Fig. 3c, PTGS1 overexpression strongly reduced ALP staining in ASCs. Consistently, Alizarin red staining revealed markedly decreased mineralization in ASCs overexpressing EGFP-PTGS1 compared with control cells (Fig. 3d). Real-time RT-PCR analysis showed impaired expression of the osteogenic marker gene *ALP* after induction in EGFP-PTGS1-overexpressing ASCs (Fig. 3e). Expression of other osteogenic marker genes including *OCN*, *RUNX2*, and *OSX* was also strongly decreased in these cells at 1 and 2 weeks after induction (Fig. 3f–h).

### PTGS1 influences osteogenesis of ASCs in vivo

**Fig. 4:**
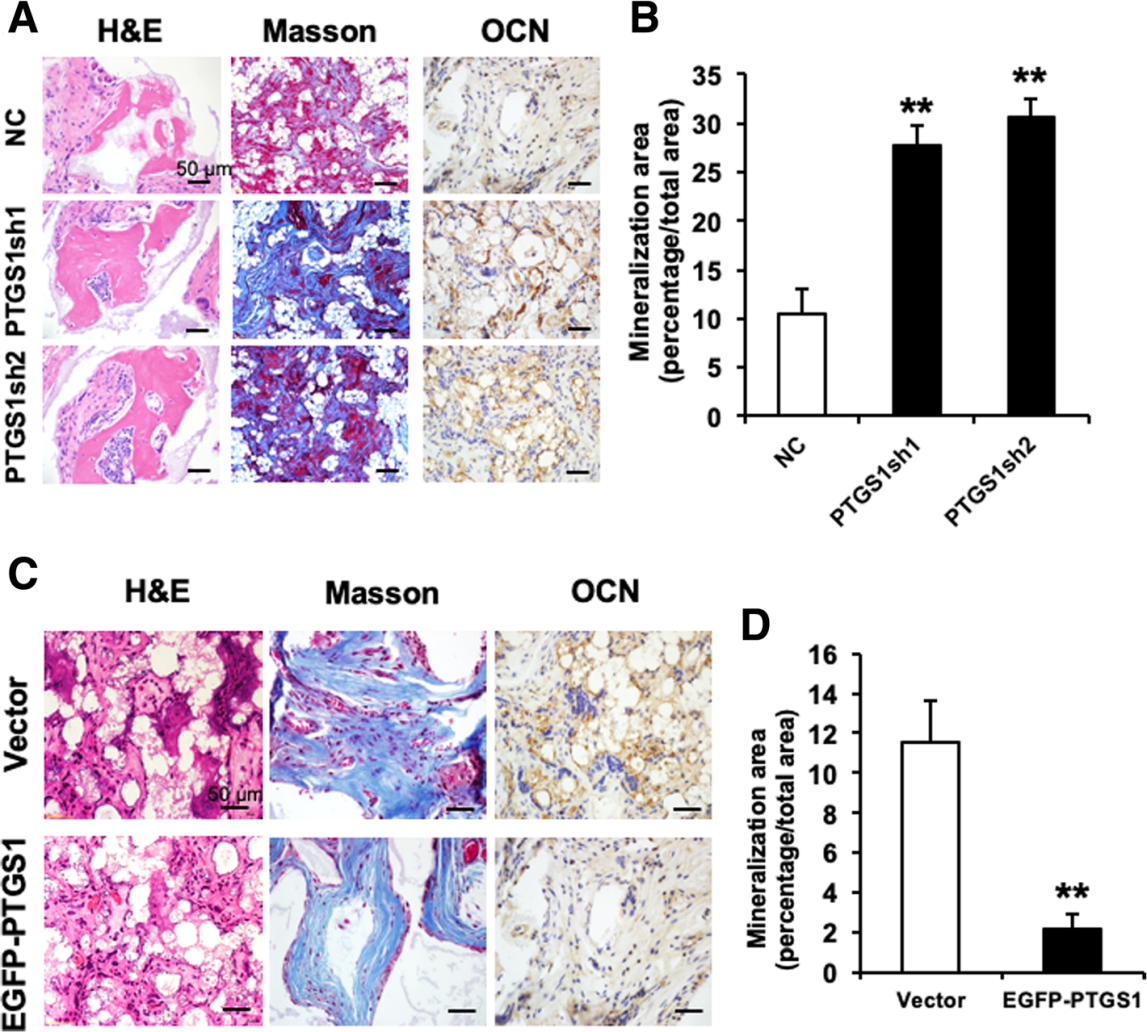
PTGS1 affects osteogenesis of ASCs in vivo*.* **a** PTGS1 knockdown promoted ASC-mediated bone-like tissue formation in vivo, as measured by HE staining, Masson’s trichrome staining, and immunohistochemistry staining of OCN in *PTGS1*sh and NC groups. Scale bar represents 50 μm. **b** Quantitative measurements of bone-like tissues showed that the area of bone formation was notably increased in PTGS1 knockdown cells compared with NC cells. **c**, **d** PTGS1 overexpression impaired the osteogenic differentiation in ASCs, as shown by HE staining, Masson’s trichrome staining, immunohistochemistry staining of OCN, and quantitative measurements of bone-like tissue. Scale bar represents 50 μm. All data are shown as the mean ± SD, *n* = 3. **P < 0.01

To further study the function of PTGS1 in the osteogenesis of ASCs, we tested whether knockdown or overexpression of *PTGS1* affected ASC-mediated bone regeneration in vivo. First, PTGS1-modified cells were incubated with synthograft (matrix material) and transplanted into the dorsal space of mice. After 8-week implantation, the samples were harvested, processed, and subjected to histological analysis. First, HE staining showed that the *PTGS1*sh group generated much more bone-like tissue compared with the NC group (Fig. 4a), and quantitative analysis verified that silencing of PTGS1 greatly promoted bone-like tissue formation in vivo (Fig. 4b). Masson staining indicated increased collagen deposition in the *PTGS1*sh group compared with the NC group (Fig. 4a). The quantity and intensity of positive OCN-stained tissue was obviously increased in PTGS1*-*silenced cells (Fig. 4a). Consistently, overexpression of PTGS1 significantly attenuated the formation of bone-like tissue, as measured by HE staining, quantitative measurements, and Masson staining (Fig. 4c, d). Altogether, these results demonstrate a novel role for PTGS1 in ASC-mediated bone formation.

### Knockdown of PTGS1 inhibits the translocation of p65 from the cytoplasm to the nucleus

**Fig. 5:**
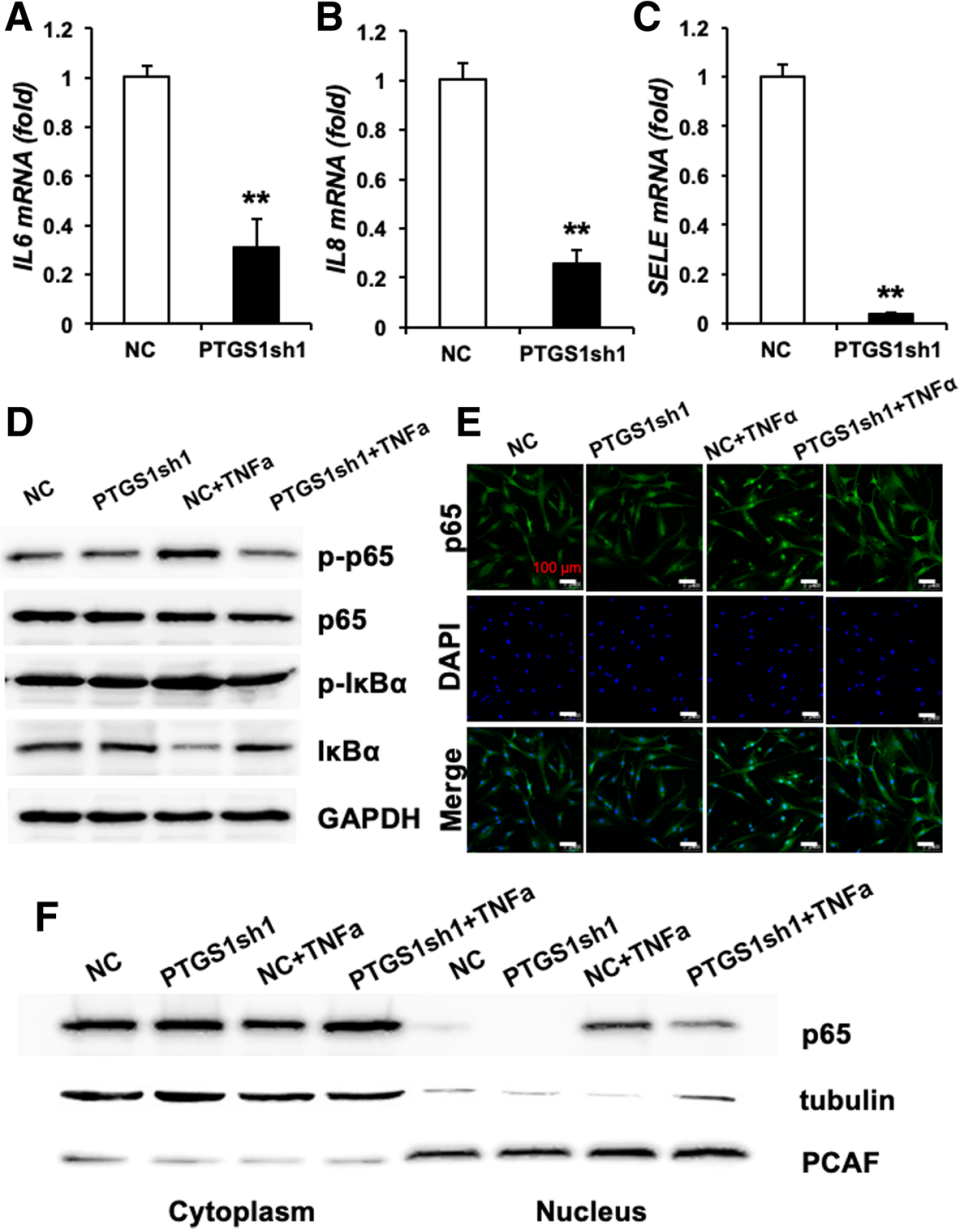
Knockdown of PTGS1 inhibits the translocation of p65 from the cytoplasm to the nucleus. **a**–**c** Real-time RT-qPCR showed that expressions of p65 target genes *IL6*, *IL8*, and *SELE* were reduced when PTGS1 was silenced with a short hairpin RNA. GAPDH was used as an internal control. **d** The protein levels of p-IκBα, total IκBα, p-p65, and total p65 were measured by Western blot in the absence and presence of TNFα for 0.5 h. **e** The cellular localization of endogenous p65 was observed by confocal microscopy in both control and PTGS1 knockdown cells with or without TNFα treatment. Scale bars, 100 μm. **f** The nuclear and cytoplasmic levels of p65, tubulin, and PCAF were measured by Western blot after subcellular fractionation from *PTGS1*sh cells untreated or treated with TNFα for 0.5 h. GAPDH was used as an internal control. All data are shown as the mean ± SD, *n* = 3. **P < 0.01. NC negative control cells, *PTGS1*sh PTGS1 knockdown cells

In order to elucidate the molecular mechanism by which PTGS1 regulates osteogenesis in ASCs, we next investigated the potential effect of PTGS1 on NF-κB, the master regulator of the inflammatory response. As shown in Fig. 5a–c, expressions of the p65 targets *IL6*, *IL8*, and *SELE* were impaired in PTGS1 knockdown cells, suggesting that PTGS1 is a positive regulator of the p65 pathway.

**Fig. 6:**
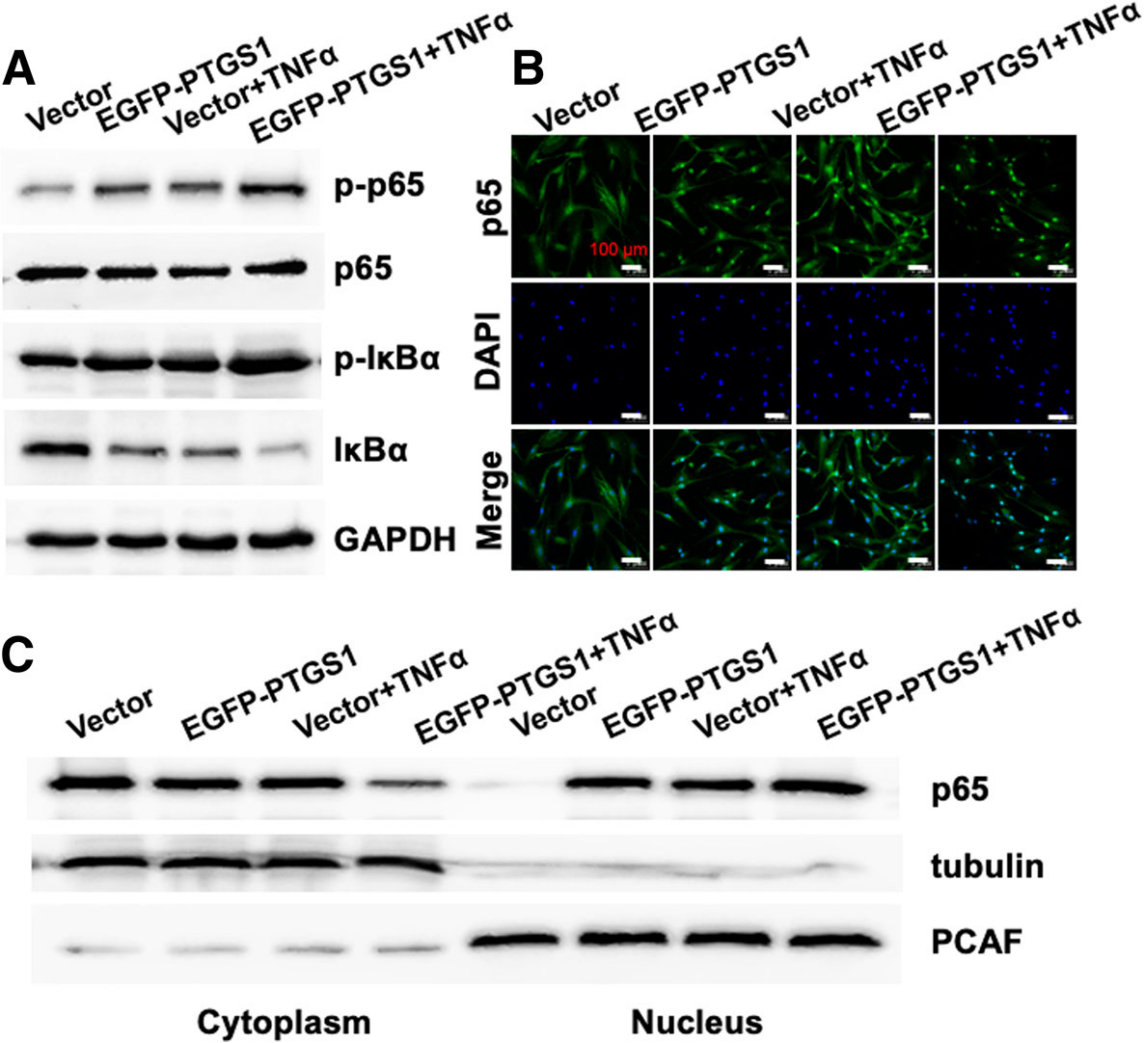
PTGS1 promotes the translocation of p65 from the cytoplasm to the nucleus. **a** The protein levels of p-IκBα, total IκBα, p-p65, and total p65 were measured by Western blot in the absence and presence of TNFα for 0.5 h. **b** The cellular localization of endogenous p65 was observed by confocal microscopy in both control and PTGS1-overexpressing cells with or without TNFα treatment. Scale bars, 100 μm. **c** The nuclear and cytoplasmic protein levels of p65, tubulin, and PCAF were measured by Western blot after subcellular fractionation from EGFP-PTGS1 and vector groups untreated or treated with TNFα for 0.5 h. GAPDH was used as an internal control. All data are shown as the mean ± SD, *n* = 3

Next, we studied whether the classical or non-classical NF-κB signaling pathway was activated during PTGS1-mediated osteogenic differentiation of ASCs. We observed that silencing of PTGS1 significantly reduced the phosphorylation and degradation levels of IκBα protein in the presence of TNFα. Moreover, the phosphorylation level of p65 was also significantly inhibited in PTGS1 knockdown cells (Fig. 5d). To further explore the underlying mechanism, we studied the subcellular localization of p65 in PTGS1 knockdown cells. Confocal microscopy results showed the nuclear translocation of p65 induced by TNFα stimulation was reduced in PTGS1 knockdown cells (Fig. 5e). Next, we prepared nuclear and cytoplasmic protein extracts, and Western blot analysis showed that silencing of PTGS1 led to reduced p65 accumulation in the nucleus (Fig. 5f). To confirm the role of PTGS1 in the regulation of nuclear translocation of p65, we examined the status of the NF-κB signaling pathway in PTGS1-overexpressing cells. Consistently, our results indicated that overexpression of PTGS1 markedly increased the phosphorylation and degradation levels of IκBα and the phosphorylation of p65 with or without TNFα treatment (Fig. 6a). As shown in Fig. 6b, c, overexpression of PTGS1 effectively increased the nuclear exclusion of p65 as shown by Western blot and confocal microscopy analyses. Altogether, these results demonstrate that knockdown of PTGS1 inhibits the NF-κB signal through promoting the nuclear exclusion of p65. (All original western membranes: Additional file 2).

## Discussion

PTGS1 has been implicated in numerous pathophysiological processes including arthritic disease, cancer, pain, and inflammation, but its role in osteogenic differentiation and inflammatory regulation in MSCs remains fairly vague. Significant evidence has accumulated revealing that inhibition of PTGS1 yields an anti-inflammatory effect. Previous research showed that genetic deletion or pharmacological inhibition of PTGS1 could reduce the LPS-induced inflammatory response, potentially acting through the NF-κB pathway. *Ptgs1*-deficient mice showed a decreased level of arachidonic acid-induced inflammation [12, 23].

In this study, we found that PTGS1 was activated upon TNFα treatment in ASCs. Moreover, deletion of PTGS1 substantially attenuated the expressions of *IL6*, *IL8*, and *SELE*. These results indicate that inhibition of PTGS1 may have the potential to reduce inflammation in ASCs. Previously, the role of PTGS1 in osteogenesis has rarely been documented. A related study reported that the PTGS1 inhibitor BGJb can rescue bone resorption induced by IL1 [15]. Herein, we demonstrated knockdown of PTGS1 significantly enhanced the osteogenic differentiation of ASCs, and overexpression of PTGS1 inhibited osteogenesis capacity both in vitro and in vivo. This is consistent with the above report, which indicated PTGS1 exerts a negative regulatory role in bone formation. Research about bone forming related to blocking inflammation is a meaningful part for bone tissue regeneration, which was closely coordinated by cell microenvironment. Bone injury model with inflammation is needed to carry out in our future research.

We found that PTGS1 expression is highly induced in response to TNFα treatment and that the inflammatory cytokine could inhibit osteogenesis and bone formation by downregulating two core transcription factors for osteogenic differentiation: RUNX2 and OSX [24, 25]. TNFα is the key stimulating factor for activating the classical NF-κB pathway; for this reason, we hypothesized that targeting the NF-κB pathway may be a novel mechanism through which PTGS1 regulates the osteogenic differentiation of MSCs. For NF-κB family, the p50-p65 heterodimers remain segregated in the cytoplasm through interaction with IκBa. After stimulation by TNFα, the classical pathway is triggered as follows: IKK complexes are phosphorylated by the IκB kinase, IκBa protein undergoes phosphorylation and ubiquitin-dependent degradation, and the p50-p65 dimers are released from IκBa protein, translocating to the nucleus. In addition, the nuclear p50-p65 dimer can also combine with IκBa protein to form a strong nuclear export signal. The heterodimer is crucial for the expression of genes encoding pro-inflammatory mediators and anti-osteogenesis regulators [26, 27].

Mechanistically, we found that knockdown of PTGS1 substantially repressed the expressions of NF-κB target genes (*IL6*, *IL8*, and *SELE*). Furthermore, deletion of PTGS1 reduced the phosphorylation and degradation levels of IκBa protein and the phosphorylation of p65. Conversely, overexpression of PTGS1 could induce the phosphorylation and degradation levels of IκBa protein and the phosphorylation of p65. These results reveal that inhibition of PTGS1 exerts pro-osteogenic function in ASCs by regulating the classical NF-κB pathway. Furthermore, we also found that *PTGS1* gene silencing can prevent nuclear translocation of p65, while its overexpression resulted in accelerated nuclear entrance of p65, as verified by confocal microscopy and Western blot analyses of nuclear and cytoplasmic cell fractions. The new link between PTGS1 and NF-κB suggests their collaboration in regulating the osteogenic differentiation and anti-inflammation of ASCs; future research is needed to further define the underlying mechanism.

In summary, our study demonstrates that knockdown of PTGS1 promotes the osteogenic differentiation of ASCs both in vitro and in vivo by targeting p65 cytoplasmic/nuclear translocation. More importantly, silencing of PTGS1 may provide a potential means for modulating the inflammatory microenvironment during bone remodeling. Our findings demonstrate that PTGS1 can be a potential target for treating inflammation-associated bone defects and is a key factor to consider in stem cell-based approaches for bone tissue regeneration.

## Conclusions

Our results first demonstrated that inhibition of PTGS1 promoted the osteogenic differentiation of ASCs through repressing NF-κB pathway by targeting p65 cytoplasmic/nuclear translocation. Furthermore, knockdown of PTGS1 may provide a potential means for regulating inflammatory microenvironment in the process of bone remodeling. Collectively, PTGS1-NF-κB signaling pathway is a novel molecular target for ASC-mediated regenerative medicine.

## Additional files

Additional file 1: **Figure S1.** Knockdown of PTGS1 enhances the osteogenic differentiation in vitro. A Microscopic images of GFP-positive ASCs under light and fluorescence microscopy. Scale bar, 500 μm. B-C Knockdown of PTGS1 was verified by real-time RT-qPCR and Western blot. D-E PTGS1 knockdown promoted ALP activity and increased ALP staining. F-G PTGS1 knockdown increased mineralization, as shown by Alizarin red staining and quantitative calcium analysis. H-L Silencing of PTGS1 increased the expressions of *ALP*, *OCN*, *BSP*, *RUNX2*, and *OSX*. **P < 0.01. NC negative control cells, *PTGS1*sh PTGS1 knockdown cells, d day, w week. (TIFF 2714 kb)

Additional file 2: **Figure S2.** All original western membranes. (TIFF 2886 kb)

## Abbreviations

ALP: Alkaline phosphatase
ARS: Alizarin red staining
ASCs: Adipose-derived stem cells
BSP: Bone sialoprotein
GAPDH: Glyceraldehyde-3-phosphate dehydrogenase
HE: Hematoxylin and eosin
IHC: Immunohistochemistry
IL6: Interleukin 6
IL8: Interleukin 8
MSCs: Mesenchymal stem cells
NF-κB: Nuclear factor kappa B
OCN: Osteocalcin
OSX: Osterix
PTGS1: Prostaglandin G/H synthase 1
RUNX2: Runt-related transcription factor 2
SELE: E-selectin
TNFα: Tumor necrosis factor alpha
