# Supplementary figures and images for: Inhibition of PTGS1 promotes osteogenic differentiation of adipose-derived stem cells by suppressing NF-kB signaling
Source: Stem Cell Res Ther. 2019 Feb 13;10:57. doi: 10.1186/s13287-019-1167-3 (PMC6375160; doi:10.1186/s13287-019-1167-3)

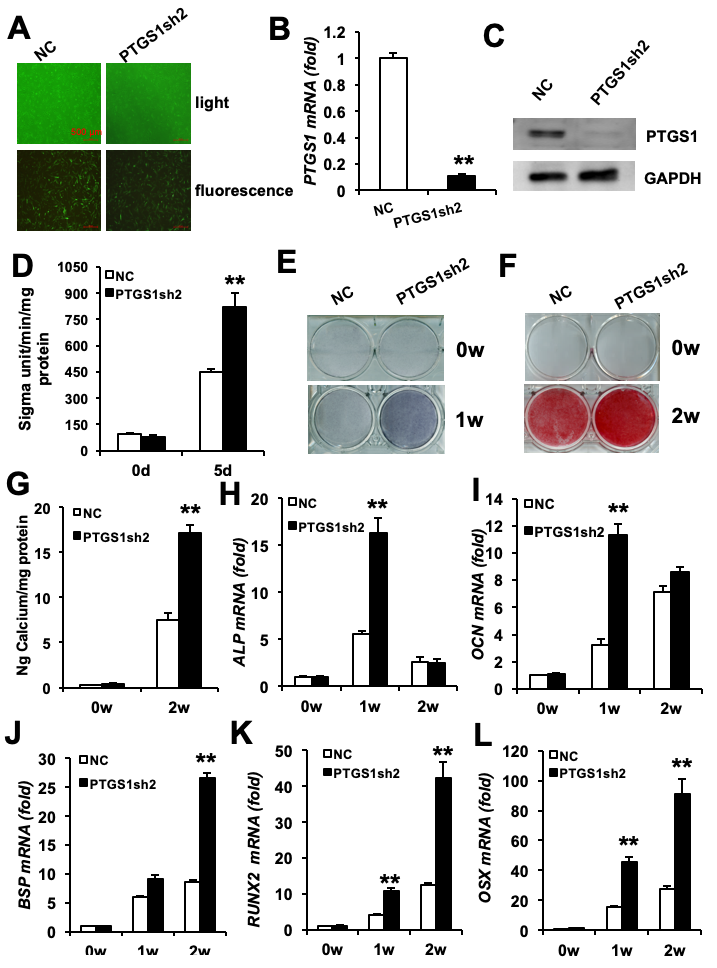

Supplement: Supplementary file 1 — Figure S1. Knockdown of PTGS1 enhances the osteogenic differentiation in vitro. A Microscopic images of GFP-positive ASCs under light and fluorescence microscopy. Scale bar, 500 μm. B-C Knockdown of PTGS1 was verified by real-time RT-qPCR and Western blot. D-E PTGS1 knockdown promoted ALP activity and increased ALP staining. F-G PTGS1 knockdown increased mineralization, as shown by Alizarin red staining and quantitative calcium analysis. H-L Silencing of PTGS1 increased the expressions of ALP, OCN, BSP, RUNX2, and OSX. **P < 0.01. NC negative control cells, PTGS1sh PTGS1 knockdown cells, d day, w week. (TIFF 2714 kb) [file 13287_2019_1167_MOESM1_ESM.tiff]

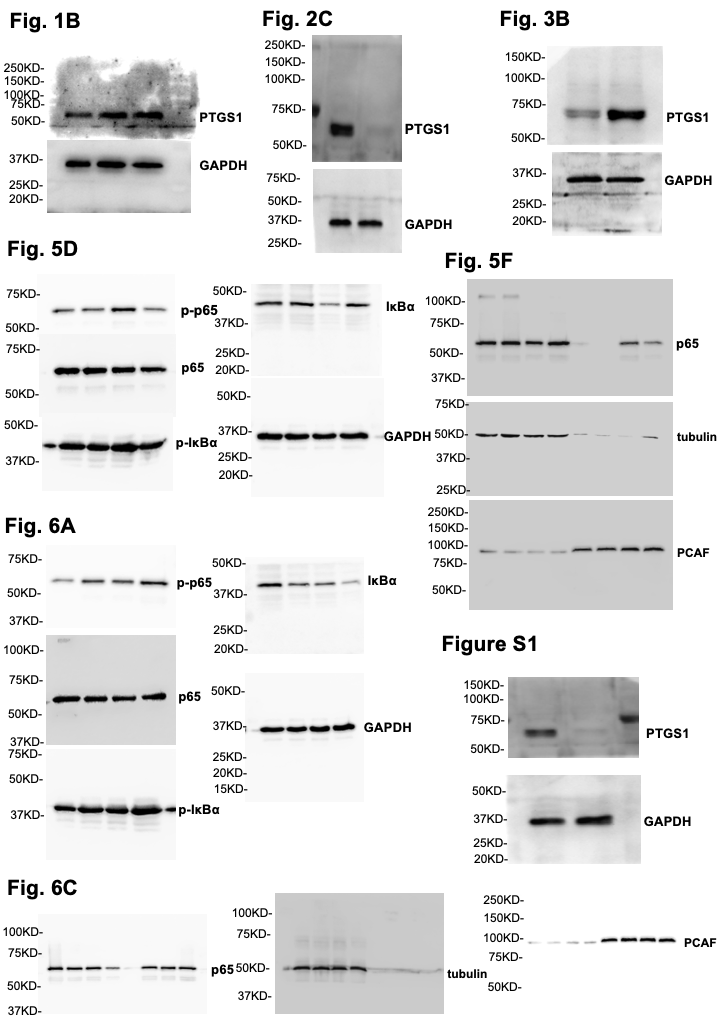

Supplement: Supplementary file 2 — Figure S2. All original western membranes. (TIFF 2886 kb) [file 13287_2019_1167_MOESM2_ESM.tiff]
